# Sustainability Assessment of a District-Wide Quality Improvement on Newborn Care Program in Rural Rwanda: A Mixed-Method Study

**DOI:** 10.5334/aogh.3205

**Published:** 2021-04-19

**Authors:** Evrard Nahimana, Hema Magge, Francois Bizimana, Merab Nyishime, Christina Thompson Lively, Hannah Gilbert, Bethany Hedt Gauthier, Felix Sayinzoga, Fulgence Nkikabahizi, Lisa R. Hirschhorn

**Affiliations:** 1 Partners In Health/Inshuti Mu Buzima, Kigali, Rwanda; 2 Bill and Melinda Gates Foundation, USA; 3 Division of Global Health Equity, Brigham and Women’s Hospital, Boston, USA; 4 Department of Global Health and Social Medicine, Harvard Medical School, USA; 5 Rwanda Biomedical Center, Kigali, Rwanda; 6 Kinihira Hospital, Rwanda Ministry of Health, Kigali, Rwanda; 7 Northwestern University Feinberg School of Medicine, USA

## Abstract

**Background:**

Neonatal mortality continues to be a global challenge, particularly in low- and middle-income countries. There is growing work to reduce mortality through improving quality of systems and care, but less is known about sustainability of improvements in the setting post initial implementation. We conducted a 12-month sustainability assessment of All Babies Count (ABC), a district-wide quality improvement project including mentoring and improvement collaborative designed to improve quality and reduce neonatal mortality in two districts in rural Rwanda.

**Methods:**

We measured changes in key neonatal process, coverage, and outcome indicators between the completion of ABC implementation and 12 months after the completion. In addition, we conducted 4 focus group discussions and 15 individual in-depth interviews with health providers and facility and district leaders to understand factors that influenced sustainability of improvements. We used an inductive, content analytic approach to derive six themes related to the ABC sustainability to explain quantitative results.

**Findings:**

Twelve months after the completion of ABC implementation, we found continued improvements in core quality, coverage, and neonatal outcomes. During ABC, the percentage of women with 4 antenatal visits increased from 12% to 30% and remained stable 12 months post-ABC (30%, p = 0.7) with an increase in facility-based delivery from 92.6% at the end of ABC to 95.8% (p = 0.01) at 12-month post-ABC. During ABC intervention, the 2 districts decreased neonatal mortality from 30.1 to 19.4 deaths per 1,000 live births with maintenance of the lower mortality 12 months post-ABC (19.4 deaths per 1,000 live births, p = 0.7). Leadership buy-in and development of self-reliance encouraging internally generated solutions emerged as key factors to sustain improvements while staff turnover, famine, influx of refugees, and unintended consequences of new national newborn care policies threatened sustainability.

**Interpretation:**

Despite discontinuity of key ABC support, health facilities kept the momentum of good practices and were able to maintain or increase the level of prenatal, neonatal quality of care and outcomes over a period of 12 months following the end of initial ABC implementation. Additional studies are needed to determine the longer-term sustainability beyond one year.

## Introduction

Over the past two decades, countries across the globe have made substantial improvements reducing under-five mortality overall, yet 3 million newborn deaths and 2.6 stillbirths [1] still occur every year. Most (99%) of these deaths occur in low- and middle-income countries (LMICs) where high neonatal mortality rates are often associated with poor quality of maternal and neonatal care services [1,2]. Most of these deaths could be avoided with simple and affordable evidence-based interventions [3,4].

To achieve the health-related sustainable development goal (SDG) of reducing preventable deaths of newborns to at most 12 per 1,000 live births by 2030, high burden countries must effectively and sustainably implement evidence-based interventions in maternal and newborn care that could reduce neonatal deaths by as much as 71% annually [5,4]. Governments and their partners are currently implementing programs to achieve these goals, but little is known of how these improvements can be sustained beyond the intervention period and factors related to their short- and long-term sustainability. Understanding these factors and implementation strategies is critical for policy makers, program designers, and funders as they seek to ensure communities will continue to benefit after projects end.

**Figure 1:**
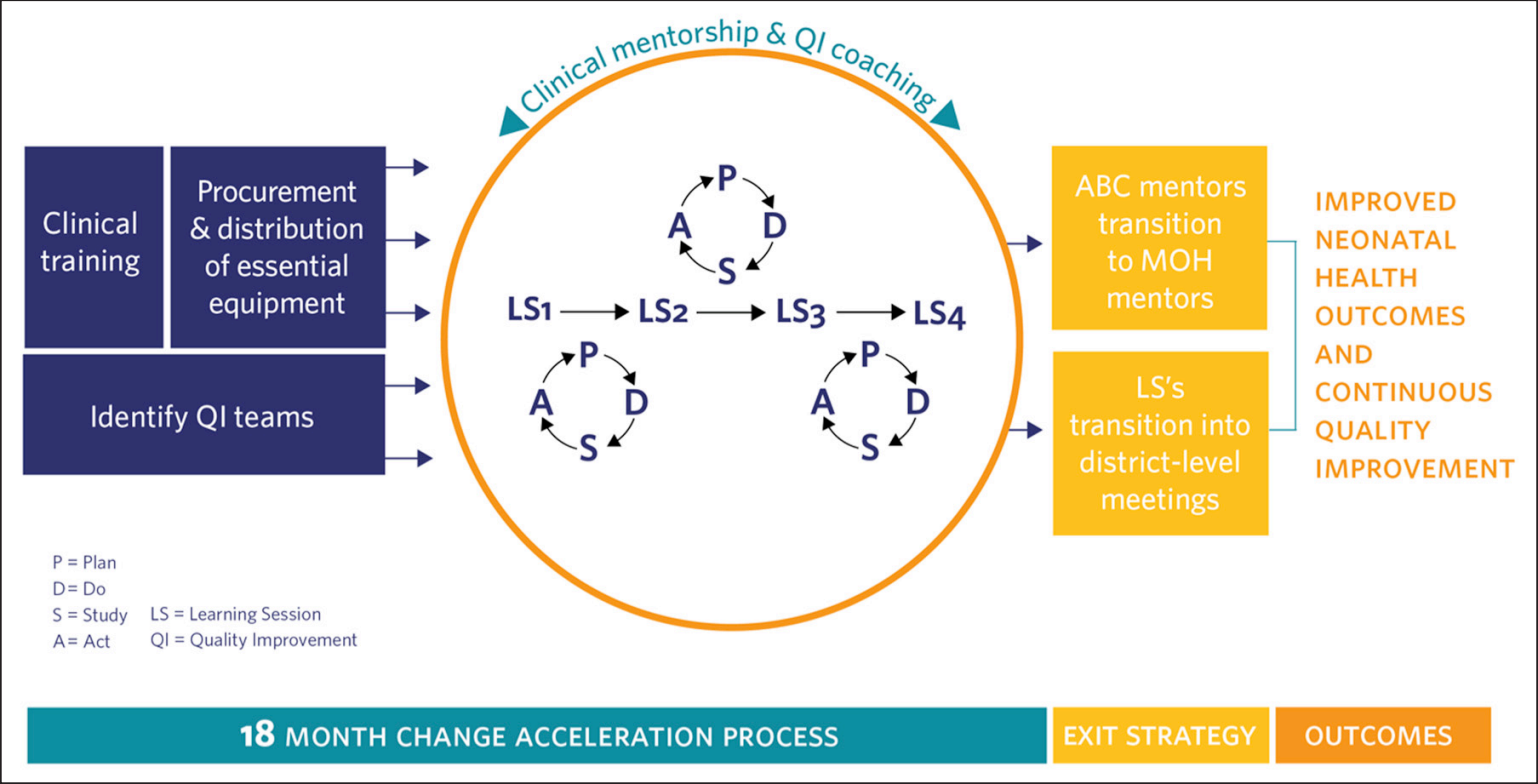
ABC Conceptual Model.

Despite impressive progress over the past decade reducing under-five mortality, neonatal mortality in Rwanda remains high at 20 newborn deaths per 1,000 live births [6]. Given high rates of facility delivery and that 90% of neonatal deaths occur within the first 48 hours after birth, facility-focused interventions will play a critical role in reducing neonatal mortality. The Rwandan government, together with Partners In Health/Inshuti Mu Buzima (PIH/IMB), a non-profit organization working in Rwanda since 2005, designed and implemented “All Babies Count” (ABC), a district-wide quality improvement program to eliminate preventable neonatal deaths in two districts in the Eastern Province of the country [7,8]. The ABC intervention package included neonatal care provider trainings, limited equipment support, clinical mentorship and quality improvement (QI) coaching, and the establishment of district-wide QI learning collaboratives (***Figure 1***). The ABC program was successfully implemented in all health facilities across the 2 districts (Kirehe and S. Kayonza), including 24 health centers and 2 district hospitals [7]. A pre-post evaluation of ABC showed significant improvement in multiple measures of antenatal, delivery, and postnatal quality of care and in district neonatal mortality [7].

In this paper, we describe the work to integrate key elements of the ABC program into routine systems and the results of a mixed-methods study to evaluate 12 months sustainability of improvements seen during the ABC program. We also explored factors related to the success and challenges of sustainability from the perspective of key stakeholders, including health providers, mentors, and local leaders.

## Methods

### Study Setting and Context

ABC was implemented between July 2013 and September 2015 in two districts, Kirehe and Southern Kayonza (S. Kayonza), located in Eastern Province of Rwanda. The two districts have been partnered with PIH/IMB and the Rwanda MOH to support health systems since 2005 in all 24 health centers (16 in Kirehe and 8 in S. Kayonza) and 2 district hospitals (1 in each district). The two districts serve a population of more than a half million. In Kirehe, three health centers (Kigarama, Rwantonde, and Mahama) were opened after the launch of ABC in October 2013. In S. Kayonza, one health center (HC) (Rwinkwavu HC) does not provide maternity services so was excluded from some indicators, such as number of pregnant women with antenatal care visits and number of deliveries. During ABC implementation, efforts were made to integrate selected ABC activities, including neonatal care mentorship and peer-to-peer learning, into existing district routine activities. No additional equipment, supply, or training related to neonatal care were given to facilities after the 12-month completion of ABC by PIH/IMB, and all health facilities in the two districts continued to be managed by the MOH.

### Study Design

We used a mixed-methods approach with convergent sequential design to study the sustainability of improvements and ABC activities 12 months after the completion of the ABC program (September 2015) and factors related to the success and challenges of sustainability from the perspective of key stakeholders including health providers, mentors, and local leaders. This included a quantitative evaluation using a pre-post design to capture changes in key processes, coverage, and neonatal outcomes from the end of ABC to 12 months post-intervention. We also conducted focus group discussions and in-depth interviews with health workers at the facilities, ABC team members, and local leaders to understand which activities sustained and which ones dropped. We captured their experiences and opinions on neonatal practices, how they changed over time after the ABC implementation phase, and factors they thought were important to increase or threaten sustainability of improvements and best practices.

### Data Collection and Sampling

Quantitative: Indicators used for measurement were the same as those used during the ABC program and for the primary evaluation [7]. These were selected based on the globally accepted process and impact outcomes for maternal and newborn health and were aligned with Rwandan priorities and context. Three time periods (three months each) were considered in our analysis: 1) Baseline (pre-ABC), July to September 2013; 2) ABC Endpoint July to September 2015 (9–12 months of ABC interventions); and 3) 9–12 months post-intervention end (July to September 2016).

Data were extracted from the existing national and district data management systems facility (Health Management Information System for Facility Data (HMIS)) and community health worker (CHW) (Système D’Information Sanitaire Communautaire (SIS-Com)). We used patient charts from facilities and records from CHWs to validate data from HMIS and SIS-Com respectively. In case of differences between the two data sources, priority was given to facility and community health records as the primary source. To complement routinely collected facility data, ABC program monitoring data were used for indicators not reported in HMIS or SIS-Com. Facility surveys conducted during the ABC program were also repeated to measure availability of essential equipment and medications.

Qualitative: Four qualitative focus group discussions (FGD) with eight participants each were conducted in Kirehe and S. Kayonza with nurses and midwives providing maternal or neonatal care at health centers and nurses providing maternal or neonatal care at hospitals. Participants were purposely selected based on their experience and active participation in the neonatal and maternity services (at least two years). A facilitator and note-taker were hired and trained to lead the discussions.

Semi-structured individual interviews were conducted with 20 participants, including 2 ABC mentors, 4 MOH mentors, 1 program director, 6 nurses (3 from each district including 2 from health centers and 1 from hospitals), 4 directors of health centers, 1 district hospital director, 1 political leader, and 1 data manager. Recorded interviews occurred at a place that ensured privacy. For both focus group discussions and interviews, questions focused on the following topics: (1) experiences with ABC program; (2) challenges faced during implementation; (3) views of impact on patients; and (4) experiences during the 12 months after implementation. Focus group discussions and interviews were conducted in the local language of Kinyarwanda, were audio-recorded with permission, and were transcribed, then translated into English for data analysis.

### Analysis

Quantitative analysis: Monthly data were aggregated for each of the three time periods and analyzed across both districts and individually. Point estimates were calculated for each indicator at each time period, and change that occurred over time was analyzed. Wilcoxon signed rank test was used to test the significance of differences for continuous variables where possible. Improvement was measured from baseline to ABC endpoint, and sustainability was measured from ABC endpoint to 12 months post-ABC completion. Excel and Stata v.14 (College Station, TX: StataCorp LP) were used respectively for data management and analysis. A p-value <0.05 was considered significant.

Qualitative analysis was inductive and employed a content analytic approach [9]. A subset of transcripts was open coded for the purposes of developing a codebook; the resultant codebook was then used to direct code all transcripts. Coded data were examined inductively to derive emergent themes that were developed into conceptual categories that describe either key barriers or facilitators of ABC implementation and sustainability. Each category consisted of a descriptive label, an operational definition, and key illustrative quotes. These initial categories were reviewed and revised to create a set of six final themes that appear in the Results section below.

After independently analyzing the quantitative and qualitative data, content areas represented in both data sets were identified, and all the results were compared, contrasted, and synthesized. The separate results were then interpreted. The themes from focus group discussions and individual interviews were assessed in terms of their ability to explain quantitative results.

### Ethics/Institutional Review

All participants for the qualitative assessment provided written consent. The research was reviewed and approved by the Institutional Review Board at Harvard Medical School (HMS) (Ref No IRB16-0584) and the Rwanda National Ethics Committee (Ref. No. 895/RNEC/2016) the National Health Research Committee, a review board of the Rwanda Biomedical Center and both Kirehe and Rwinkwavu District Hospital management teams.

## Results

### Changes in post-ABC activities and performance

Twelve months following the completion of ABC, Kirehe and Kayonza District health leadership sustained selected key ABC activities although at lower intensity (***Table 3***). Mentorship was sustained but with a decrease in frequency, from an average of 1 mentoring visit per month in S. Kayonza at the end of ABC to 0.8 visit per month—with a similar drop in Kirehe (0.8 visit per month to 0.4). Peer-to-peer learning through learning sessions as well as regular review of key neonatal data were incorporated into routine monthly coordination meetings. Despite this decrease, the improvements seen in most key quality and coverage indicators during ABC were sustained and/or increased 12 months after the completion of ABC (see ***Table 2***).

The availability of essential medicine and functioning equipment to deliver newborn care remained high in both districts despite no further direct supplies from PIH/IMB. In Kirehe, the availability of essential medications, which had increased during ABC (from 67% to 78%, p = 0.04) kept increasing significantly 12 months after the end of ABC (83%, p = 0.003) (***Table 1***). The percent of women giving birth at a health facility, already high in both districts at the end of ABC (92.6%), kept increasing significantly 12 months after the end of ABC (95.8%, p = 0.01). Similarly, the percentage of pregnant women who completed four antenatal care (ANC) visits remained stable across the two districts. Aggregated across both districts, improvements were seen in labor management and newborn care after birth as we compare the end of ABC and 12 months later: steroid administration for preterm labor (41.7% to 58%, p = 0.01), systematic monitoring of danger signs for newborn 24 hours after birth (98.7% to 99.3%, p = 0.7). The percentage of babies with immediate skin-to-skin after delivery, already high at the end of ABC (97.4%), reduced slightly (96.2%, p = 0.2).

**Table 1:** Changes in Availability of Essential Medications and Equipment for Kireha (16 facilities) and Southern Kayonza (9 facilities).


INDICATOR	ABC BASELINE (JUL–SEP 2013) MEDIAN (IQR)	ABC ENDPOINT (JUL–SEP 2015) MEDIAN (IQR)	12 MONTHS POST INTERVENTION (JUL–SEP 2016) MEDIAN (IQR)	p-VALUE*

Percent availability of essential medications for maternal/newborn Care				
Kirehe	67 (55–78)	78 (73–82)	83 (83–91)	<0.01
S.Kayonza	35 (25–45)	83 (75–87)	87 (75–100)	0.39

Percent availability of functioning equipment essential for maternal/newborn care				
Kirehe	55 (48–64)	81 (77.8–89)	89 (89–94)	<0.01
S. Kayonza (N = 9)	55 (47–56)	89 (89–94)	89 (89–94)	0.4


* p-values are for comparisons of end of ABC versus 12 months post-ABC.

**Table 2:** Changes in Healthcare Coverage and Quality between the end of ABC and 12 months post-ABC.


INDICATOR	AGGREGATE					SOUTH KAYONZA					KIREHE			
		
	BASELINE (JUL–SEPT 2013)	ABC ENDPOINT (JUL–SEPT 2015)	12 MONTHS POST-ABC (JUL–SEPT 2016)	P-VALUE COMPARING 12 MONTHS POST-ABC TO ABC ENDPOINT		BASELINE (JUL–SEPT 2013)	ABC ENDPOINT (JUL–SEPT 2015)	12 MONTHS POST-ABC (JUL–SEPT 2016)	P-VALUE COMPARING 12 MONTHS POST-ABC TO ABC ENDPOINT		BASELINE (JUL–SEPT 2013)	ABC ENDPOINT (JUL–SEPT 2015)	12 MONTHS POST-ABC (JUL–SEPT 2016)	P-VALUE COMPARING 12 MONTHS POST-ABC TO ABC ENDPOINT
		
	MEDIAN (IQR)	MEDIAN (IQR)	MEDIAN (IQR)			MEDIAN (IQR)	MEDIAN (IQR)	MEDIAN (IQR)			MEDIAN (IQR)	MEDIAN (IQR)	MEDIAN (IQR)	

Percent of deliveries with mothers with 4 or more ANC visits	13.7 (6.7– 44.2)	30.4 (15.1–43.1)	30.9 (23–47)	0.7		42.4 (19.9–47.3)	36.4 (13.5–66.3)	53 (24–100)	0.3		10.2 (2.8–26.8)	29.6 (18–34.2)	30.7 (20–37)	0.04

Percent of pregnant women delivering in facilities	89.6 (86.3–94.9)	92.6 (86.8–95.8)	95.8 (93–97.4)	0.0148		92.2 (87.2–95.3)	95.2 (93–96.3)	96.1 (95.7–98.3)	0.092		88.4 (86.3–96.7)	91.0 (86.2–94.6)	94.3 (91–97.2)	0.06

Percent of babies provided immediate skin-to-skin after birth	53.6 (0–80.9)	97.4 (96.4–99.3)	96.2 (86–100)	0.2		91.2 (75.9–100)	99.6 (96.7–100)	94.2 (87.7–96.3)	0.05		4.3 (0–53.6)	97.2 (96.1–98.8)	96.5 (85.8–100)	0.8

Percent of newborns checked for danger signs within 24 hrs of birth	46.6 (31.1–96.7)	98.7 (96.4–100)	99.3 (89–100)	0.7		52.3 (33.8–98.6)	100 (93.8–100)	96.2 86.8–98.8	0.01		45.7 (15.8–82.1)	98.6 (97.4–98.8)	100 (98.2–100)	0.4

Percent women with preterm labor who are treated with antenatal steroids	0 (0–0)	41.7 (0–100)	58 (48–67)	0.02		0 (0–0)	16.7 (0–33.3)	54.5 (48.2–86)	0.03		0 (0–0)	75 (0–100)	66.6 (62–78)	0.4


**Table 3:** Examples of Ideas for improving the quality of neonatal care that were continued 12 Months after the end of ABC.


	INDICATOR TARGETED	CARE GAP	CHANGE CONCEPT

**Antenatal Care**	Percent of Women with 4 ANC visits	4 ANC visit completion is low because women miss their first ANC appointment	Test women for pregnancy in all departments and transfer for ANC enrollment or same day care if pregnant

		No mechanism to follow up with women who miss appointments	Make (a) a filing system of medical records or (b) a register modification such that women who miss appointments can be easily recognized and contacted by CHWs

		Women don’t come for ANC because partner is not available	See women for 1st ANC and send her with invitation for her partner to attend following visit

**Delivery Management**	Percent of pregnant women delivering in facilities	Women do not deliver at the facility because they fail to do appropriate anticipatory planning	Assist mothers with anticipatory planning of items to have prepared to bring for delivery at their 3rd ANC appointment

	Percent of women with pPROM treated with antibiotics	Low case identification of women in active preterm labor	Refresher trainings for staff on calculation of gestational age and management of pPROM to improve recognition of labor complications and management

	Time to cesarean section	Poor communication and coordination between maternity and neonatal services	Set aside a regularly scheduled time for collaboration between neonatal and maternity services

**Post-natal care**	Percent of newborns checked for danger signs within 24 hours of birth	Low maternal knowledge and nurse vigilance in checking for neonatal danger signs	Low maternal knowledge and nurse vigilance in checking for neonatal danger signs


Similarly, neonatal mortality dropped from 30.1 deaths per 1,000 live births at the beginning of ABC to 19.4 deaths per 1,000 live births. The decrease in mortality was maintained 12 months post-ABC at 19.4 deaths per 1,000 live births (p = 0.7). Building capacity and ownership for QI was also a goal of ABC. At the end of ABC, there were 47 active QI projects towards improving maternal and neonatal care across both districts. While there was a decrease in number, 12 months post-ABC, QI projects were still being implemented. All facilities (100%) in S. Kayonza and 60% in Kirehe were implementing at least one QI project per health center. In addition, a number of key improvements initiated by health center teams during the ABC period continued 12 months later (***Table 1***).

### Qualitative Findings

Six major themes linked to the success or challenges of sustainability of ABC and related improvements were inductively identified. Quotes reflecting each theme are summarized in ***Table 4***.

**Table 4:** Key themes associated with facilitating and challenging factors to ABC sustainability of activities and improvements.


THEMES	EXAMPLES OF QUOTES

Leadership “Buy-In” and “Ownership”	“During the second learning collaborative in Kirehe, the mayor, the vice mayor and the entire leadership team were present. They stayed for the whole day. They took notes and asked questions” I was surprised to see the Mayor sitting there for hours. And you can see that he was very interested and curious to see what is happening. We were feeling supported.” – ABC mentor “…Kabarondo titulaire is a nun. She also a student. She is doing her bachelor degree in nursing, so she is very busy. One day I went to Kabarondo for mentorship and found the maternity register not completed and showed the maternity team what is missing. I gave feedback to the team and the titulaire was there. Two weeks later, when I went back there, I found everything in place. The titulaire despite her busy schedule, took time to fix that herself. You understand that she cares about the work. She did not delegate, she did the work, because she cares.” – ABC mentor

Benefits and Drawbacks of Young Leadership	“My impression is that you have a country with a relatively young leadership. Relatively young in the sense of it’s a maturing leadership. On one hand, you have this complexity of an incredibly ambitious Ministry of Health at the highest level of leadership. On the other hand, you have different layers of young leaders, who are dynamic but with lack of managerial and technical experience.” -ABC program coordinator “…Rwanda is a young nation. From its background of genocide and civil war, they had to build everything from scratch. We did a lot of things from 1994, almost miracles. But you understand that we are still learning how to build this country.” – Political Leader, Kirehe

Losing Trained Staff as a Key Barrier to Sustainable Improvements	“…There are HC like Rwantonde, Kabuye and Gahara, there are factors behind. Normally there were some key persons at the health center we were used to work together. During mentorship, we were working with everyone, but they are specific people that were driving the improving. Unfortunately, most of these people left. They are no longer at the HC either because of the issue of documents (diplome) or just they left because they found another job.” – ABC mentor “This was an error to build capacity on just one person. This was the case in Kabuye Gahara and in other HC. For other Health centers, you can find nurses who have been working in that department(maternity) for many years, so they bring a new staff, it is a problem because he/she will start from scratch.” – ABC mentor

Unplanned Adverse Events Go with Unexpected Great Opportunities to have an Impact on Neonatal Outcomes.	“I think [The change in ANC coverage] makes totally sense (changes in antenatal care coverage). If you look the baseline and during ABC, there was no famine yet or maybe it was not much. But it got worsened in the end of 2015 and 2016 which is the period of sustainability. Maybe the drought started a bit early, but people started to feel it in that period. And it was terrible. I know people who flee the country to Tanzania and Uganda because of the famine.” – Health center nurse “There was a time, there were few pregnant women coming in our health center for antenatal care visit. And when we asked community health workers, they explained that people are very hungry because of the drought. Husbands prefer to flee the country to find jobs. They leave their wives and children home. It is difficult to go to travel to health center when you are hungry and alone at home.” – Nurse from Health center in S. Kayonza “At Kirehe hospital, normally we receive between 150 to 200 of women in maternity per month. But when Burundian refugees came in, we were overwhelmed with pregnant women with more than 300 of admissions per month. Most of the women came from Mahama.” – Nurse at Kirehe hospital

Building Self-reliance, Encourages internally Generated Solutions	“In Ndego, the health center worked with CHWs to identify women who missed their appointment- to sensitize them to return to health center for follow-up. They also talk to the women in the village to understand why they don’t return. This has happened in Ruramira too. Even though there were some quality issue but they had a register where they document women who missed their appointment to track them later. They used to call them. This helped a lot to increase the number of women who come for antenatal care visit.” – ABC mentor

Gap Between High Demand in Maternal and Neonatal Services and Adequate Human Resources	“If the hospital leadership knew deliveries went up from 200 to 300 in a month because the flux of refugees with only 12 nurses in maternity working day and night for 7 days a week, they should be able to recruit more nurses to adjust. Otherwise, what are you expecting for the 12 nurses to do in front of the increasing demand? Same for neonatology, a lot has been done during intensive phase and mortality went down significantly. There was a time 1 nurse alone had to manage 30 sick babies. Of course, mortality will go high. So, if deliveries went high, the number of sick babies will go high too, and the number of babies with birth asphyxia too. So, I think if the hospital can’t take measures to hire more personnel you understand there is an issue. So here I may say that health providers did not do a good job, but it is not their fault. The problem is they were overwhelmed.”- Health center Nurse in Kirehe


*Leadership “buy-in” and “ownership*” (Facilitator): This emerged as one of the powerful factors to facilitate various activities aimed at improving the quality of neonatal care. Participants not only defined leadership as those occupying top positions of political or administrative authority, but also extended the concept of leadership to include any person across the continuum of care whose engagement and enthusiasm pushed forward the neonatal care agenda. Participants noted the presence and active participation of district authorities during learning collaborative sessions and coordination meetings after ABC period.

*Young leadership* (Facilitator and Challenge): Interviewees described how national, district, and local leaders were ambitious, optimistic, confident, and committed, which was both a facilitator and barrier. Leaders were described as committed and ready to do whatever it takes to reduce neonatal mortality. However, most were young and often lacked managerial and technical experience. This combination of ambition and lack of managerial expertise meant that they were easily pulled in many directions, and participants explained that this led to a loss of focus on neonatal health, which affected the momentum after the ABC period.

*Turnover of trained staff* (Challenge): The mobility of staff was identified as an important challenge to sustainable improvements. Health care workers who chose to leave did so for multiple reasons. During the implementation phase of ABC, selected nurses and midwives at the hospital and health centers were trained in maternal and neonatal care services. As they became skilled and experienced, they became more attractive to other health facilities, particularly those in urban areas. The departure of the trained staff for other health facilities left their previous facilities with critical staff shortages, which negatively impacted services.

*Stuff happens!* (Challenge): Both unplanned adverse events and unexpected great opportunities have an impact on neonatal outcomes. Participants described unexpected events that impacted neonatal outcomes both over the course of ABC implementation and during the one year post-intervention period. These events were not anticipated during the design of the ABC program and could not have been predicted, so stakeholders were not always prepared to respond to them. The events included a prolonged drought causing famine in Eastern province, the influx of Burundian refugees in Kirehe District, unintended consequences of new policies from district or central level, such as introduction of an antenatal care fee, which decreased the number of women attending antenatal care services.

*Development of self-confidence fostered internally generated solutions* (Facilitator): Participants noted the emergence and importance of locally made solutions to improve antenatal care services, delivery management, and post-natal care. Innovative, individualized ideas that could be integrated into ABC practices at each site were generated by health centers and hospital teams with the support of ABC mentors. These internally generated solutions started during the ABC implementation period and continued in the year that followed the end of the program. They took different forms depending on the health facility. These included 1) partnering with community health workers (CHWs) and local leaders to identify pregnant women and encourage them to attend ANC visits or 2) improving internal communication across different services within the health center, 3) motivating women to attend health centers for antenatal care and deliveries by providing non-financial incentives, such as clothes, to the pregnant women who attended the center during the first ANC visit during their first trimester.

*Gap between high demand in maternal and neonatal services and adequate human resource* (Challenge): Interviewees explained that staff shortages and work overload made it difficult for staff to meet expected goals for newborn care services, a barrier to having achieved and sustained improvements. Community demand steadily increased, and a growing number of women began seeking care at the health centers or hospitals. Almost all pregnant women came to deliver at the health center or hospital. Unfortunately, human resources did not increase to match this demand, which negatively impacted the quality of services provided to pregnant women.

## Discussion

Despite decreased direct support and challenges, core components of ABC were sustained, and health facilities continued to initiate and/or maintain existing QI projects and sustainability or improvement of improvements in maternal and neonatal performance measures. We identified both program design and external contextual factors from key informants that contributed to the resilience of the improvement. These included strong leadership and ownership as well as confidence and ability in developing locally made solutions. We also identified factors that emerged as challenges to sustaining the progress and sustainability of the ABC-associated progress, such as the influx in refugees in Kirehe and the famine following a prolonged drought. Despite these challenges, the improvement associated with ABC were also sustained in key process and system quality, coverage, and outcome indicators.

While there is a wealth of literature discussing sustainability of public health programs [10,11], we did not find any that explore sustainability after the end of a QI intervention to improve systems in neonatal care quality in limited-resource settings. Our findings on sustainability of improvements may have been related to the design and implementation strategies, which were consistent with factors associated with sustainability from the literature. ABC program was intentionally designed to include a number of these important strategies, such as engagement with a range of key stakeholders (Ministry of Health authorities, district leaders, representative from health facilities through regular joint meetings) throughout the project or combination of trainings, supervision/mentorship, and learning collaboratives, as well as strong and ongoing local community engagement. For example, a systematic review of a wide range of evidence-based programs showed that a program is likely to be sustained if it reported greater community engagement, communication with key stakeholders, knowledge of the program’s logic model by the stakeholders, and sustainability planning [11,12]. In addition, strategies targeting improving performance of health care providers are likely to produce sustained outcomes if they combine training, supervision, and group problem solving [13].

The ABC program also demonstrated flexibility to be adapted over time in response to new factors, a strategy also identified as a factor influencing sustainability of improvements [14]. Dickson and colleagues assessed health-systems bottlenecks and strategies to accelerate high impact interventions to reduce neonatal mortality in 13 high-burden countries. While the study did not focus on sustainability of programs, the recommendations for effective program implementation (including dynamic leadership, community empowerment, and capacity building for rural health workforce to upgrade relevant clinical skills for care) were shared with the design of ABC and our findings of factors associated with sustainability of the ABC impact [15].

Our study contains a number of limitations. The absence of comparison groups from other districts in Rwanda with similar settings does not allow us to make any inference on the sustainability compared with other districts’ neonatal quality and mortality. The specific findings may also have limited generalizability to settings outside rural districts and Rwanda; however, the broader principles are likely applicable to other interventions and settings. While there is no commonly accepted time point for defining when a program is sustained, we can only report on findings at 12 months for this study.

In conclusion, we found sustainability at 12 months of the key process improvement including supply chain and care delivery as well as mortality improvements. Key implementation strategies identified as supporting the sustainability and consistent with the literature included stakeholder engagement throughout planning and implementation, intentional integration of core components of the ABC into MOH roles and function, and building capacity for QI. Additional research is needed to further understand longer-term sustainability of improvement activities and quality and how to ensure the needed resources and strategies are integrated into QI initiatives and maintain the advances critical to continue to reduce neonatal mortality.
